# Supplementary material for: Potential of Coccolithophore Microalgae as Fillers in Starch-Based Films for Active and Sustainable Food Packaging
Source: Foods. 2023 Jan 23;12(3):513. doi: 10.3390/foods12030513 (PMC9914559; doi:10.3390/foods12030513)
Supplement: Supplementary file 1 [file foods-12-00513-s001.zip › foods-2029990-supplementary.pdf]

Supplementary data

Article

Potential of Coccolithophore Microalgae as Fillers in Starch-Based Films for Active and Sustainable Food Packaging

Ana S. P. Moreira [1,2], Joana Gonçalves [3], Francisco Sousa [3], Inês Maia [4], Hugo Pereira [5], Joana Silva [5], Manuel A. Coimbra [2], Paula Ferreira [3] and Cláudia Nunes [3,*]

[1] CICECO - Aveiro Institute of Materials, Department of Chemistry, University of Aveiro, Campus Universitário de Santiago, 3810-193 Aveiro, Portugal

[2] LAQV-REQUIMTE - Associated Laboratory for Green Chemistry of the Network of Chemistry and Technology, Department of Chemistry, University of Aveiro, Campus Universitário de Santiago, 3810-193 Aveiro, Portugal

[3] CICECO, Department of Materials and Ceramic Engineering, University of Aveiro, Campus Universitário de Santiago, 3810-193 Aveiro, Portugal

[4] CCMAR - Centre of Marine Sciences, University of Algarve, Campus de Gambelas, 8005-139 Faro, Portugal

[5] GreenCoLab - Associação Oceano Verde, University of Algarve, Campus de Gambelas, 8005-139 Faro, Portugal

* Correspondence: claudianunes@ua.pt

Supplementary Table S1. Elemental analysis and protein estimation from freeze-dried biomass of *E. huxleyi* (EHUX) and *C. pseudoroscoffensis* (CP) microalgae.

**Samples**	**%C**	**%H**	**%N**	**%S**	**%CHNS**	**Protein (N × 4.78)**[c]
E. huxleyi (EHUX)	27.3 ± 0.5	3.7 ± 0.1	3.4 ± 0.0	*t*	34.4 ± 0.6	16.1 ± 0.1
EHUX (other batch)[a]	20.3 ± 0.2	4.3 ± 0.1	3.2 ± 0.0	1.3 ± 0.3	29.0 ± 0.6	15.3 ± 0.1
C. pseudoroscoffensis (CP)	21.3 ± 0.2	2.9 ± 0.1	2.3 ± 0.1	*t*	26.5 ± 0.2	11.2± 0.3
CP (other batch)[b]	22.6 ± 0.3	3.4± 0.2	2.4 ± 0.1	*t*	28.5 ± 0.6	11.6 ± 0.4

t, traces (<0.5%); [a]Data from other batch of *E. huxleyi* biomass produced under the same culture conditions [1]; [b]Data from other batch of *C. pseudoroscoffensis* biomass produced under the same culture conditions [2]; [c]Nitrogen-to-protein conversion (4.78) proposed for microalgae [3].

Supplementary Table S2. TGA values at the second stage of thermal decomposition of starch films (control) and starch-based films containing 20% of commercial $CaCO_3$, *E. huxleyi* (EHUX) or *C. pseudoroscoffensis* (CP)

**Samples**	**T_{max} (ºC)**	**Remaining ash (%)**
Starch (control)	295.8	8.0
Starch+$CaCO_3$ 20%	302.7	19.7
Starch+EHUX 20%	251.4	18.6
Starch+CP 20%	278.8	12.5

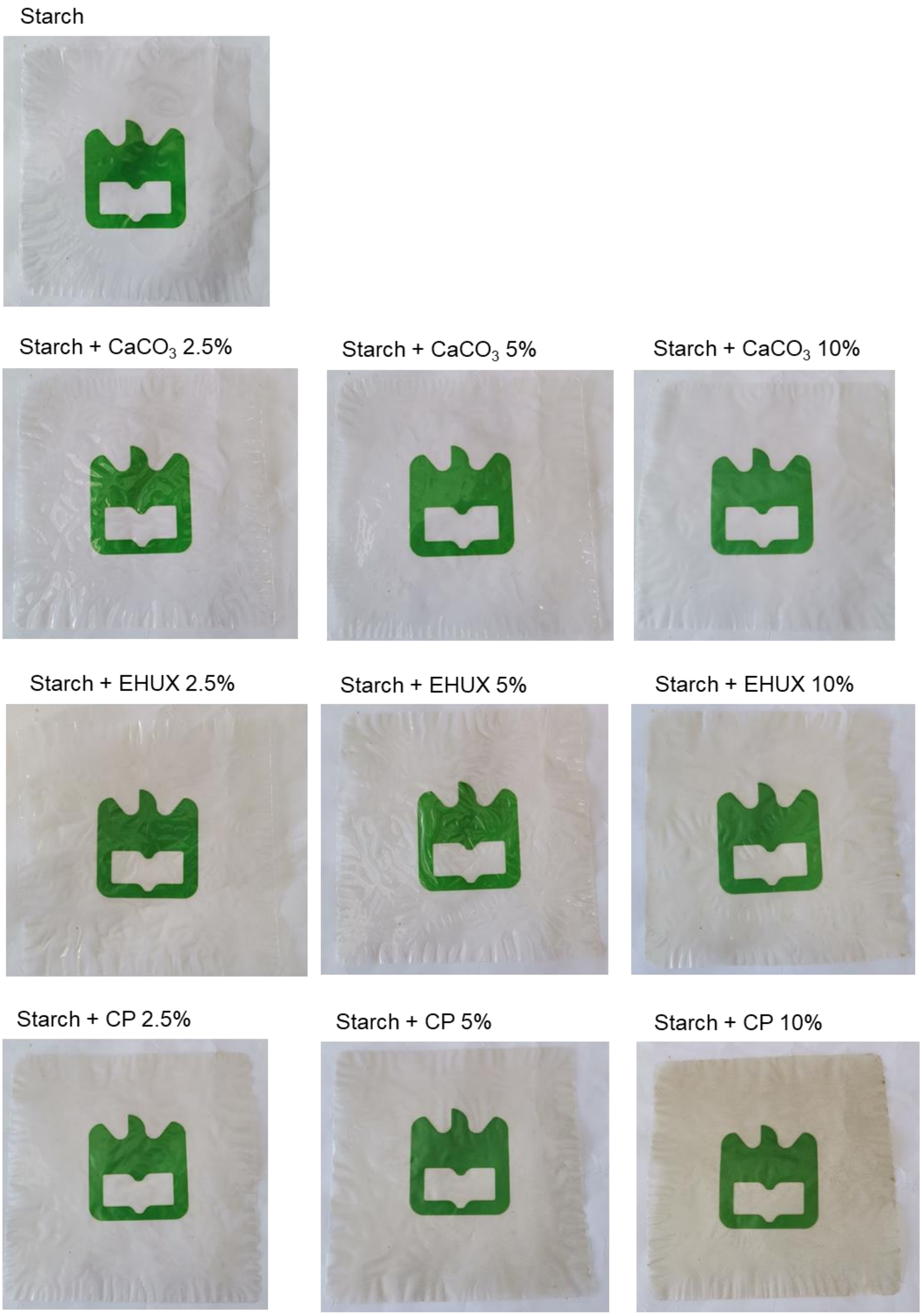

Supplementary Figure S1. Real images of starch-based films without and with 2.5, 5, and 10% (w/w of dry starch weight) of commercial $CaCO_3$, *E. huxleyi* (EHUX) and *C. pseudoroscoffensis* (CP) biomass.

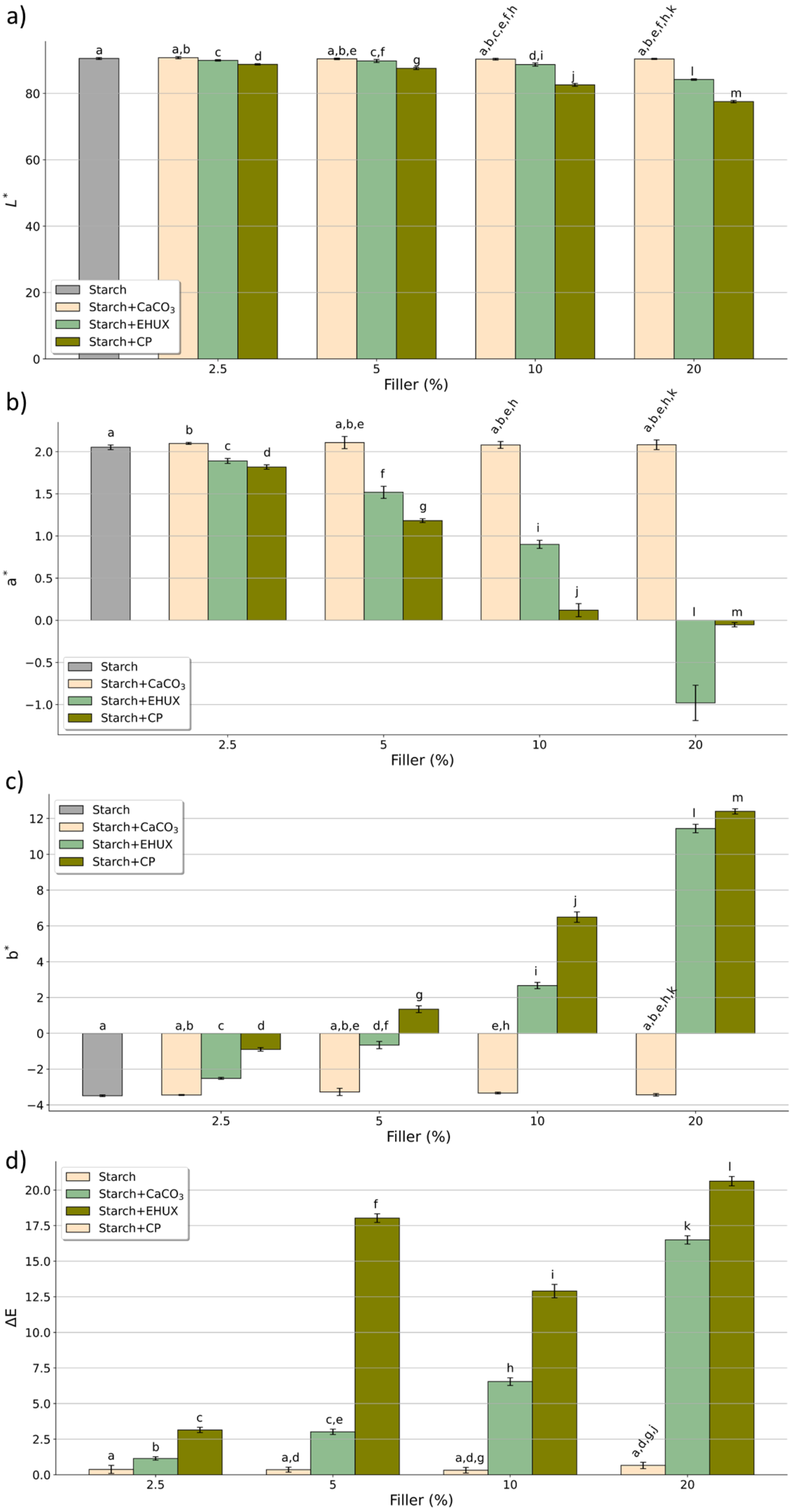

Supplementary Figure S2. Values (mean ± standard deviation) for lightness (*L**), red-green (*a**), yellow-blue (*b**), and total colour variation (ΔE) of pristine starch films (control) and starch-based films containing commercial $CaCO_3$, *E. huxleyi* (EHUX) or *C. pseudoroscoffensis* (CP) at different percentages (2.5, 5, 10 and 20% w/w). Different letters between each condition indicate significant differences (Student's t-test; $p < 0.05$).

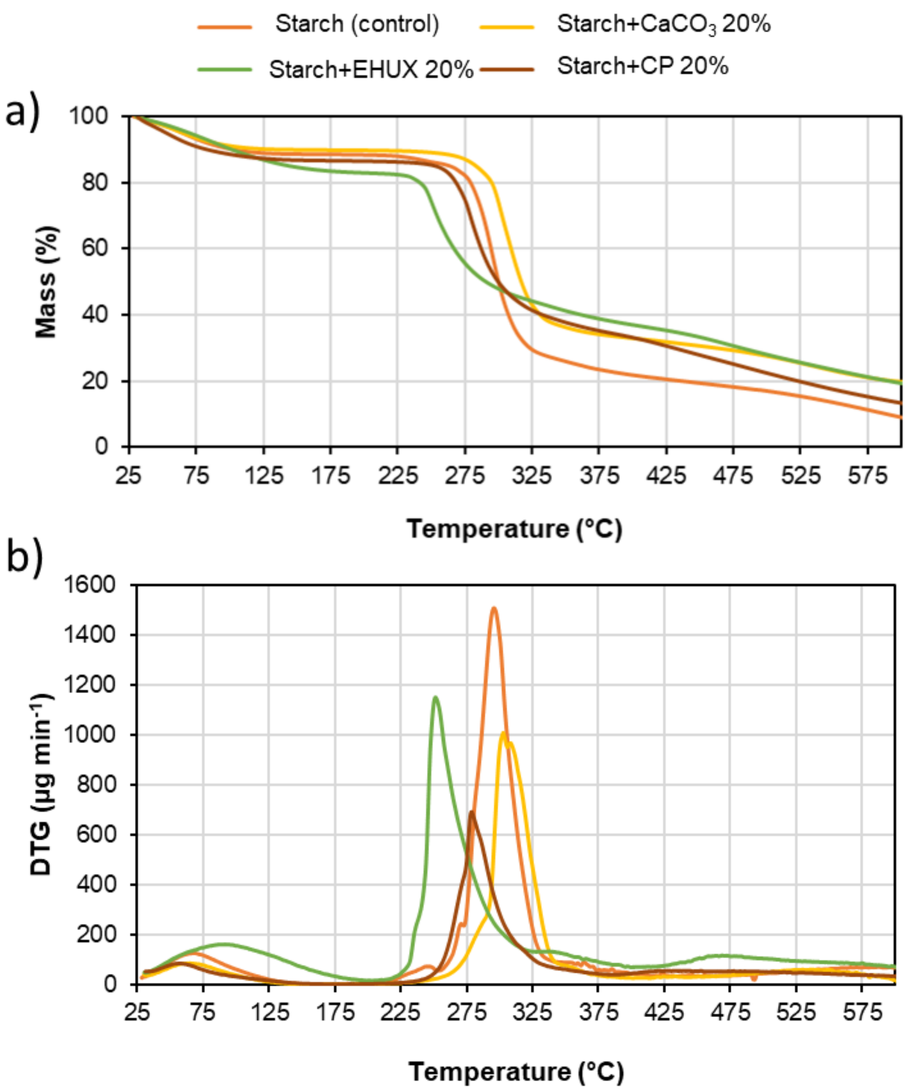

Supplementary Figure S3. a) TGA curves and b) first derivatives of starch films (control) and starch-based films containing 20% of commercial $CaCO_3$, *E. huxleyi* (EHUX) or *C. pseudoroscoffensis* (CP).

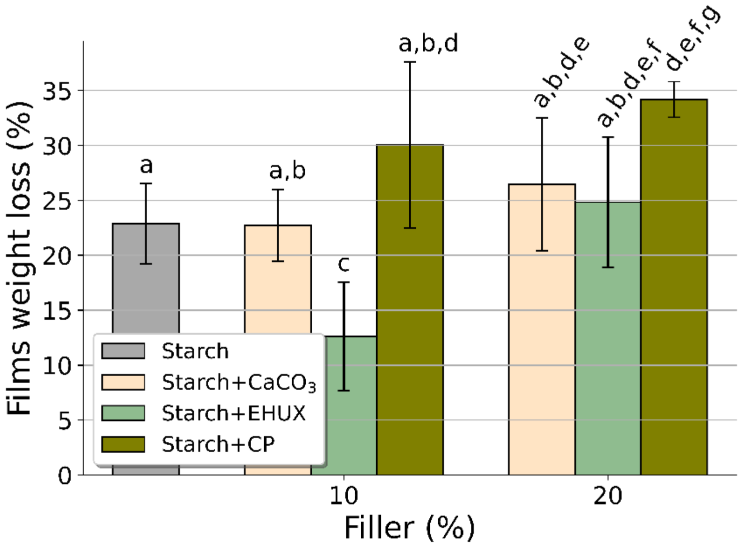

Supplementary Figure S4. Weight loss percentage observed after immersion in water for 8 days of pristine starch films (control) and starch-based films containing commercial $CaCO_3$, *E. huxleyi* (EHUX) or *C. pseudoroscoffensis* (CP) at different percentages (10 and 20% w/w). Different letters between each condition indicate significant differences (Student's t-test; $p < 0.05$).

References:

[1] S.S. Aveiro, T. Melo, A. Figueiredo, P. Domingues, H. Pereira, I.B. Maia, J. Silva, M.R. Domingues, C. Nunes, A.S.P. Moreira, The polar lipidome of cultured *Emiliania huxleyi*: A source of bioactive lipids with relevance for biotechnological applications, Biomolecules 10 (2020) 1434.

[2] A.S.P. Moreira, J. Gonçalves, T.A. Conde, D. Couto, T. Melo, I.B. Maia, H. Pereira, J. Silva, M.R. Domingues, C. Nunes, *Chrysotila pseudoroscoffensis* as a source of high-value polar lipids with antioxidant activity: A lipidomic approach, Algal Res. 66 (2022) 102756.

[3] S.O. Lourenço, E. Barbarino, P.L. Lavín, U.M. Lanfer Marquez, E. Aidar, Distribution of intracellular nitrogen in marine microalgae: Calculation of new nitrogen-to-protein conversion factors, Eur. J. Phycol. 39 (2004) 17-32.
